# A Novel Organ Culture Model to Quantify Collagen Remodeling in Tree Shrew Sclera

**DOI:** 10.1371/journal.pone.0166644

**Published:** 2016-11-21

**Authors:** Sarah Baldivia, Alexander Levy, Shylaja Hegde, Stijn J. A. Aper, Maarten Merkx, Rafael Grytz

**Affiliations:** 1 Department of Biomedical Engineering, University of Alabama at Birmingham, Birmingham, Alabama, United States of America; 2 Department of Vision Sciences, University of Alabama at Birmingham, Birmingham, Alabama, United States of America; 3 Department of Biomedical Engineering and Institute for Complex Molecular Systems (ICMS), Eindhoven University of Technology, Eindhoven, The Netherlands; 4 Department of Ophthalmology, University of Alabama at Birmingham, Birmingham, Alabama, United States of America; Cardiff University, UNITED KINGDOM

## Abstract

Increasing evidence suggests that unknown collagen remodeling mechanisms in the sclera underlie myopia development. We are proposing a novel organ culture system in combination with two-photon fluorescence imaging to quantify collagen remodeling at the tissue- and lamella-level. Tree shrew scleral shells were cultured up to 7 days in serum-free media and cellular viability was investigated under: (i) minimal tissue manipulations; (ii) removal of intraocular tissues; gluing the eye to a washer using (iii) 50 μL and (iv) 200 μL of cyanoacrylate adhesive; (v) supplementing media with Ham's F-12 Nutrient Mixture; and (vi) culturing eyes subjected to 15 mmHg intraocular pressure in our new bioreactor. Two scleral shells of normal juvenile tree shrews were fluorescently labeled using a collagen specific protein and cultured in our bioreactor. Using two-photon microscopy, grid patterns were photobleached into and across multiple scleral lamellae. These patterns were imaged daily for 3 days, and tissue-/lamella-level strains were calculated from the deformed patterns. No significant reduction in cell viability was observed under conditions (i) and (v). Compared to condition (i), cell viability was significantly reduced starting at day 0 (condition (ii)) and day 3 (conditions (iii, iv, vi)). Tissue-level strain and intralamellar shear angel increased significantly during the culture period. Some scleral lamellae elongated while others shortened. Findings suggest that tree shrew sclera can be cultured in serum-free media for 7 days with no significant reduction in cell viability. Scleral fibroblasts are sensitive to tissue manipulations and tissue gluing. However, Ham's F-12 Nutrient Mixture has a protective effect on cell viability and can offset the cytotoxic effect of cyanoacrylate adhesive. This is the first study to quantify collagen micro-deformations over a prolonged period in organ culture providing a new methodology to study scleral remodeling in myopia.

## Introduction

Myopia, or nearsightedness, is the most common type of refractive error affecting over 40% of adults in the United States as of 2004 [1] and over 80% of some Asian populations [2, 3]. High levels of myopia increase the risk for blinding diseases such as myopic retinopathy, maculopathy, and glaucoma [4–8]. In myopia, the focal plane of the eye presents in front of the retina, which is usually caused by an elongated posterior scleral shell [9]. There is increasing evidence from animal studies in support of an active but unknown remodeling mechanism that elongates the posterior scleral shell to match the eye’s axial length to its optical power, producing eyes with retinal focus (emmetropia) [10–14]. The aim of this paper is to present a new organ culture system and imaging strategy based on two-photon fluorescence microscopy (2PFM) to gain insight into the remodeling mechanism that underlie scleral elongation in myopia.

The sclera is an avascular tissue [15], which surrounds the posterior eye. It serves as the principal load bearing tissue of the eye, as well as an important regulator of refractive error through a remodeling mechanism that alters the eye’s axial length. The structure of the sclera and its means of altering axial length vary between species. We used tree shrews in this study, which have a fibrous sclera that is similar to that of humans [11]. The scleral extracellular matrix (ECM) is produced and remodeled by resident fibroblasts. This scleral ECM is primarily composed of collagen type I fibrils, but also contains proteoglycans, such as aggrecan, decorin, and biglycan [10, 16]. Collagen fibrils aggregate to form interwoven layers of lamellae, which make up the bulk of the scleral structure [10].

Myopia typically develops as the eye tissues grow and remodel during childhood. We define tissue growth as a mechanism that increases the amount of tissue matter while tissue remodeling involves internal tissue deformations and restructuring of the tissue’s ECM. Experiments in mammals suggest that myopia is not caused by accelerated scleral growth but rather due to accelerated scleral remodeling that leads to scleral elongation due to unknown internal tissue deformations. Experimental myopia alters the sclera by causing: (i) scleral thinning [11, 17]; (ii) reduction in scleral dry weight (3–5%) [17–19]; (iii) lower hyaluronan and sulfated glycosaminoglycan levels [19]; (iv) upregulated enzymatic degradation [20–24]; (v) downregulated collagen type I synthesis [25]; (vi) downregulation of aggrecan [26]; and (vii) a higher creep rate [27]. McBrien at al. have reported that the scleral ultrastructure remained unchanged after short-term myopia treatment (12 days) in tree shrews [17]. Only long-term myopia treatment (> 3 months) caused a significant change in the scleral ultrastructure showing a reduction of the collagen fibril diameter, in particular, at the posterior pole [17]. This reduction in collagen fibril diameter is consistent with ultrastructural observations in high myopic human eyes [28]. Based on these findings, different mechanisms have been suggested to underlie scleral remodeling in myopia. Intraocular pressure (IOP) is higher than atmospheric pressure, subjecting the sclera to continued tensile forces. McBrien et al. have suggested that biomechanical scleral weakening leads to a creep-like elongation of the sclera and myopia at normal IOP [29]. Rada et al. suggested that biochemical alterations such as the reduction in aggrecan could make it easier for scleral lamellae to slide across each other [10]. Using multi-scale computational simulations, we have recently predicted an increase in collagen fibril crimp during myopia development and hypothesized that this may be caused by intralamellar deformations between collagen fibrils [30]. Here, we present a new methodology that allows us to investigate these different hypotheses in organ culture.

To the best knowledge of the authors, collagen remodeling, such as lamellar elongation or intralamellar deformations, have never been imaged as quantitative methods were unavailable. The main challenge to quantify collagen remodeling is its relatively slow rate. In tree shrew myopia, a significant change in refractive error, axial length, or creep rate occurs within a few days [27]. Consequently, any methodology that aims to investigate scleral remodeling must allow for prolonged imaging of living tissue over a period of days. While the role of IOP in myopia is unclear, scleral remodeling occurs while the eye is subjected to IOP. Several signaling molecules, growth factors, and proteins have been identified to participate in the signaling cascade from the retina to the scleral fibroblasts during myopia development [19, 21–24, 26, 31–34]. For these reasons, we have developed a new bioreactor to maintain both IOP and viability in tree shrew scleral shells. Existing scleral organ culture models do not account for IOP and use culture media supplemented with fetal bovine serum [35–37]. Because serum contains agents and growth factors known to stimulate the biosynthesis of matrix metalloproteinase (MMP) [38], which may alter scleral remodeling *in situ*, we aimed to optimize our culturing conditions towards maximum cell viability using serum-free culture media.

Another major challenge to quantify collagen remodeling is the need for an imaging technique that allows deep tissue imaging and tracking of micro-scale deformations in living organs over a period of days. We use 2PFM as it provides critical advantages over other techniques due to its ability to image deep inside living tissues (up to 1 mm in optimal conditions) [39, 40]. Multiphoton excitation provides optical sections from deeper within scattering specimens than confocal imaging [41]. Critical for this study is the ability of 2PFM to photobleach three-dimensionally localized patterns into fluorescently labeled tissue. Photobleached grids have been used before to quantify micro-deformations in tendon fascicles [42–44], and caudal discs [45] under acute loading. All these experiments used a confocal microscope to photobleached grids into the fluorescently labeled collagen. Due to the double cone excitation shape that is inherent to confocal microscopy, fluorescently labeled tissue will also be photobleached above and below the focal point, which limits the confocal-based photobleaching technique to track two-dimensional deformations only [42–45]. In contrast to confocal microscopy, excitation is confined to a tiny focal volume (~1 femtoliter) in 2PFM allowing us to photobleach grids, relocate them, and quantify micro-deformations in the three-dimensional space [46]. Furthermore, the localized excitation volume reduces photo-damage with improvements in expected cell viability when imaging living tissue [47].

The Merkx lab has developed protein-based fluorescent probes for the specific imaging of collagen in live tissue [48, 49]. These probes are based in the collagen binding protein CNA35 and provide *in vitro* tissue images of collagen organization with improved detail when compared with second harmonic generation (SHG) microscopy [50]. In contrast to chemical-based fluorescent probes such as 5-DTAF, CNA35 probes can be used to monitor the collagen structures in live tissue cultures over prolonged time periods [48]. We use CNA35-tdTomato [48, 49], which is a complete protein-based probe that has a high quantum yield and cross-section value when excited at long wavelengths [51], making it ideal for multiphoton fluorescent imaging in our experiments.

In this paper we present (i) a new bioreactor to culture living scleral shells of tree shrews under controlled IOP conditions; (ii) a viability study to maximize cell viability in organ culture under serum-free conditions; and (iii) an imaging strategy to quantify scleral remodeling in organ culture at the lamella- and tissue-level.

## Methods

### Bioreactor

**Fig 1:**
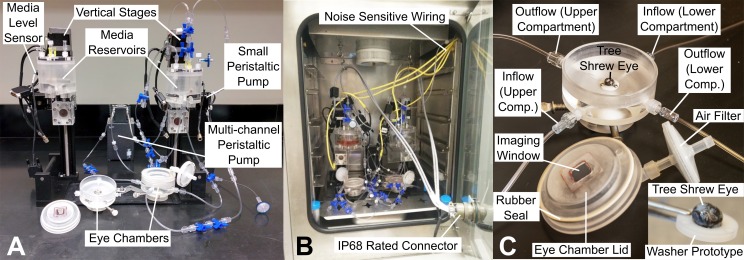
Bioreactor for tree shrew scleral organ culture and imaging. (**A**) Bioreactor for pressurizing two whole eyes glued to washers and attached to eye chambers. Vertical motorized stages are used to set the height of the media reservoirs for desired IOP through hydrostatic pressure. Two (small) peristaltic pumps (one per eye) are used for reclamation of media that may leak through the scleral shell. One multi-channel peristaltic pump is used for media circulation between the eye chambers and media reservoirs of both systems. (**B**) Bioreactor placed insight a temperature and CO_2_ controlled incubator. Most of the electrical wires used to operate the bioreactor are wired through the incubator door using an IP68 rated connecter. Noise sensitive signals are wired using individual wires (yellow) through a hole in the incubator wall. (**C**) Zoomed in view of one eye chamber. Eye chambers provide an inset for stainless steel washers with glued tree shrew eye. Underneath inset, media inflow and outflow are provided to pressurize the eye and circulate the media back to the top reservoir. Eye chamber lid provides a cover glass window for imaging sclera without exposing it to possible contaminants. Insert at the bottom right of **C** shows tree shrew eye glued to a prototype washer. Posterior sclera is on the top. Cornea was removed after the eye was glued to the washer allowing media circulation into the eye.

**Fig 2:**
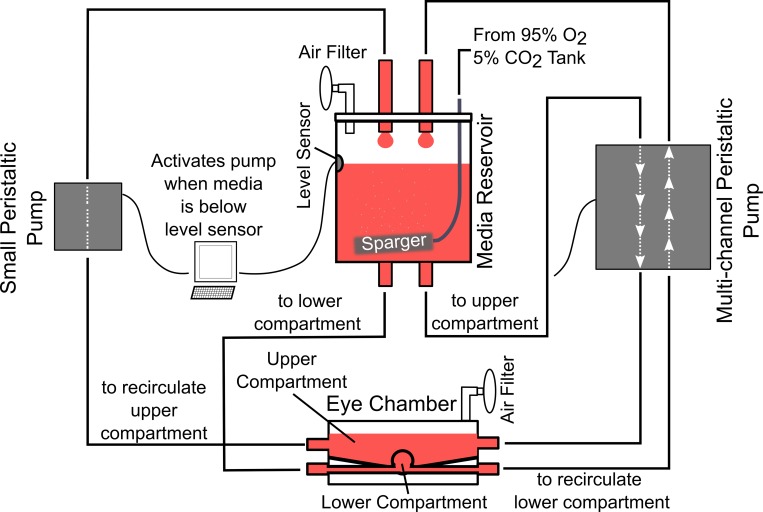
Schematic of the media flow in the bioreactor. Media from elevated media reservoir pressurizes the eye through hydrostatic pressure. A multi-channel peristaltic pump is used to circulate the media between the two compartments of the eye chamber (inside and outside the eye) and the media reservoir. To maintain a constant media level in the reservoir and to compensate for trans-scleral leakage, a small peristaltic pump is activated when media level drops below the level sensor, pumping media from the upper eye chamber compartment to the media reservoir until it reaches the sensor again.

We designed a portable bioreactor (Fig 1A) to culture and pressurize two tree shrew scleral shells at a desired IOP. The bioreactor can be placed inside a regular sized incubator to maintain optimal organ culture conditions (Fig 1B). The bioreactor was built on top of a baseplate to facilitate its transportation to the microscope for daily imaging of the remodeling scleral collagen. Each tree shrew eye is mounted to the bioreactor through a secured stainless steel washer, to which the eye is glued using a cyanoacrylate adhesive (Permabond 792) (Fig 1C). Two cannulas insert below this washer to allow media inflow and outflow for perfusion and IOP control. A ball joint at the base of the eye chamber allows for tilting of the eye chamber. Tree shrew sclera can be highly pigmented. The ball joint allows us to tilt the eye chamber and image the sclera at a different apex in case the posterior pole is highly pigmented. Eye chamber lids have been designed to allow for hermetic enclosure of eye chamber after initial placement of the scleral shell into the bioreactor. The enclosed eye chambers reduce the chance of potential contamination when the bioreactor is moved to and from the microscope for daily imaging. The eye chamber lids have a cover glass window through which the sclera is imaged without exposing the eye to potential contaminants. A constant IOP of 15 mm Hg is provided through hydrostatic pressure of media in an elevated reservoir. Within the elevated reservoir, a gas mixture of 95% O_2_ and 5% CO_2_ is bubbled to help maintain media oxygenation and pH. One multi-channel peristaltic pump (MP^2^ Micro Peristaltic Pump MP2-6-PC, Elemental Scientific, Omaha, NE, USA) is used to circulate oxygen-rich media between the elevated reservoir and the eye chamber compartments inside and outside the eye at 430 μl/min. As the sclera is permeable, media leakage occurs through the sclera during the culture period. An optical media level sensor together with a computer controlled peristaltic pump (150 series, 150-800-012-201-3, Williamson Manufacturing Co Ltd, UK) are used for each sclera shell to pump leaked media back from each upper eye chamber into the elevated reservoir assuring that a constant IOP is maintained and no media spill occurs during the culture period (Fig 2). The two scleral shells have independent media circulation systems (i.e. the culture media was not mixed between the two eyes). A pressure gauge (700G, Fluke Corporation, WA, USA) was used at the outflow port of the lower eye chamber compartment to verify the IOP level and quantify IOP fluctuations during media circulation. IOP fluctuation were less than 0.1 mmHg in our bioreactor experiments.

### Subjects

Animal studies were approved by the University of Alabama Institutional Animal Care and Use Committee (project number: IACUC-10280), and in accordance with the National Institutes of Health (NIH) guidelines for animal care. Animals were obtained from the tree shrew colony of the University of Alabama at Birmingham. Animals were housed individually in 1.52 m^3^ cages under 14/10 hours light/dark cycles with continuous access to a water bottles and dry food. Each cage had a resting board installed. The health of the animal was monitored on a daily basis. Juvenile tree shrews (*Tupaia glis belangeri)* were euthanized using a lethal injection of ketamine. After confirmed death, both eyes were enucleated and extraocular tissues were removed under sterile conditions. Tissue hydration was maintained in warm culture media. Eyes were randomly divided into the different experimental groups for the cell viability study. At least 4 eyes per group were used in the cell viability study. Two eyes of one normal juvenile tree shrew were enucleated at 35 days of visual experience and used for quantifying scleral remodeling in our bioreactor.

### Viability Study

To optimize organ culture procedures and conditions, the effect of the following variables on cellular viability was investigated: glue, removal of intraocular tissues, and culturing eyes in our bioreactor. We use high glucose Dulbecco’s modified Eagle’s medium (DMEM) (4500 mg/L) containing 25 mM HEPES and 1% Penicillin/Streptomycin (Pen/Strep) as our basic serum-free culture media. Scleral shells, with cornea removed, were cultured up to 7 days under the following six conditions:

1. whole eye with minimal tissue manipulations (positive control);
2. isolated scleral shells (cornea, vitreous, retina, choroid, and retinal pigment epithelium removed);
3. whole eye glued to a stainless steel washer using 50 μl of cyanoacrylate adhesive (Permabond 792);
4. same as condition (iii) with increased amount of glue (200 μl);
5. same as condition (iv) plus supplementing culture media with Ham's F-12 Nutrient Mixture (1:1 mixture);
6. same as condition (iv) but scleral shells were cultured in our bioreactor and subjected to 15 mmHg IOP.

The extraocular tissues were removed after the eyes were enucleated. No disinfectant was used as we processed the eyes immediately after enucleation. No further tissue manipulations were performed in condition (i) representing our positive control condition. Condition (ii) involved removing the cornea by using a razor blade; inverting scleral shells on a cotton tip to gently scrape off the retina, and choroid; and then reinverting the scleral shells. For conditions (iii-vi), the anterior segment was glued to a custom machined and flanged stainless steel washer. To allow media inflow into the eye, the central cornea was removed after the eye was glued to the washer (conditions (iii-vi)). Non-pressurized eyes (conditions (i-v)) were individually cultured in petri dishes while our bioreactor was used for condition (vi). The petri dishes (conditions (i-v)) and the bioreactor (conditions (vi)) were placed inside a temperature and humidity controlled incubator at 37°C and 95% air to 5% CO_2_ atmosphere during the culture period. For all conditions, the culture media was replaced with fresh media every other day. The number of samples varied between 4 to 8 samples per condition and culture day.

Viability was tested on days 0, 3, and 7 for non-pressurized eyes (conditions (i-v)), and on days 0 and 3 for pressurized eyes (condition (vi)). The organ culture experiments using condition (vi) were discontinued after day 3 as imaging scleral remodeling was not feasible thereafter. On these days, two punches (2 mm) were taken from each eye, stained for 10 minutes with 0.1 μM Sytox Green followed by 30 minutes staining with 3 μg/mL Hoechst 33342, and then plated between two coverslips for imaging. Sytox Green is a nuclei stain that is impermeant to live cells, making it a useful indicator of dead cells. Hoechst 33342 is nuclei stain that is permeable to live and dead cells. The tissue samples were washed 3 times with fresh culture media after each staining period. Within 2 hours after staining, the tissue punches were imaged using a confocal microscope (Nikon A1, Nikon Instruments Inc, Melville, NY, USA). Three random x-y locations were identified and 40 μm z-stacks starting 10 μm below the exterior tissue surface were imaged using 406 nm and 488 nm excitation wavelengths and optimized filter cubes. Cell nuclei within z-stacks were counted using the automated 3D object counter of NIS-Elements (Nikon Instruments Inc, Melville, NY, USA). Cell nuclei stained with Sytox Green were considered dead cells, while Hoechst stained nuclei represented the total number of cells (dead plus vital cells). We define cell viability based on a Sytox Green exclusion criterion:

$$100\;x\;\left(1-\frac{total\;number\;of\;dead\;cells}{total\;number\;of\;cells}\right)$$

The cell viability of each eye was calculated as the average viability of two punches taken from this eye and the three imaging locations per punch. Statistical significance was evaluated using two-way ANOVA with culture condition and days of incubation as fixed factors. Equal variance was determined using the Levene’s test. Post hoc pairwise analyses using Fisher’s least significant difference method were performed to evaluate a statistical significant difference between conditions (ii-vi) and the control condition (i). Finally, a pairwise comparison of the interaction term was performed to test whether or not the cell viability changed with incubation days within each condition. All statistical analyses were performed using IBM SPSS (Version 23.0. Armonk, NY: IBM Corp). A significance level of α = 0.05 was used, which was reduced to α = 0.01 when unequal variances or outliers were found.

### Imaging Scleral Remodeling

**Fig 3:**
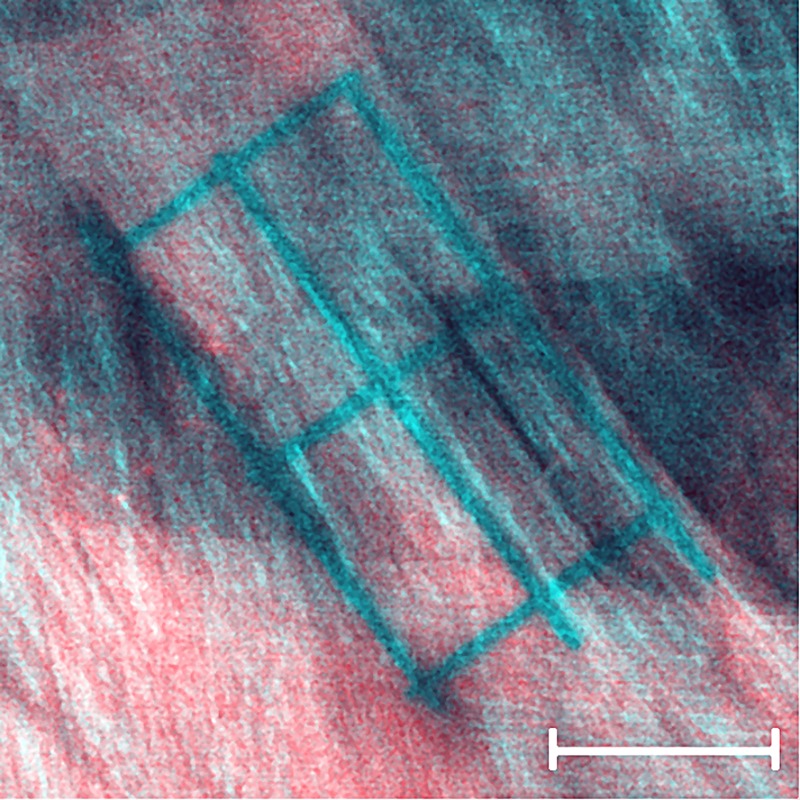
SHG image overlaid onto simultaneously imaged 2PFM image of a photobleached sclera lamella. The SHG reveals the lamella orientation showing that the grid pattern was photobleached into one lamella running from the top left corner to the bottom right corner of the image. Scale bar represents 20 μm.

**Fig 4:**
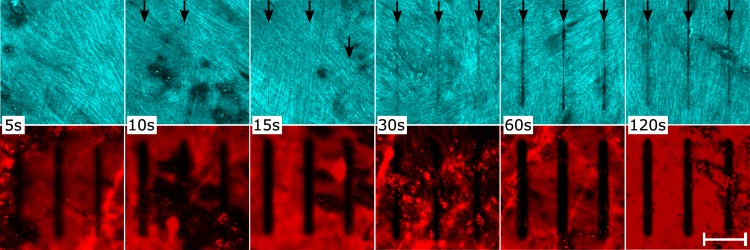
SHG and 2PFM images of scleral collagen after photobleaching at variable bleaching times. SHG (top row) and 2PFM (bottom row) images of fluorescently tagged (CNA35-tdTomato) and photobleached tree shrew sclera. A set of three grid lines were photobleached using 50% laser power and 5, 10, 15, 30, 60, 120 seconds exposure time (left to right). SHG images revealed that photobleaching for 30 seconds and more caused obvious damage to the collagen structure. Minimal damage was seen after 10 and 15 seconds while no damage was found when photobleaching for 5 seconds. Black arrows indicate observable damage to collagen fibrils caused by photobleaching. Scale bar represents 20 μm.

Two tree shrew eyes were used for imaging scleral remodeling in organ culture. The tissues were processed according to culture condition (iv). The extraocular tissues were removed and each eye was glued to a washer (Fig 1A) immediately after enucleation. The cornea was removed after the eye was glued to the washer to allow media circulation into the eye. The eyes were stained in culture media supplemented with 1 μM CNA35-tdTomato for 18 hours. CNA35-tdTomato was expressed in E.coli and purified using a single-step Ni-affinity purification as previously described [49]. After staining, eyes were mounted into our bioreactor and subjected to 15 mmHg IOP. To reduce the dissociation of the fluorescent protein, the eyes were cultured in media supplemented with a low concentration of CNA35-tdTomato. The culture media consisted of high glucose DMEM containing 25 mM HEPES, 1% Pen/Strep, and 0.01 μM CNA35-tdTomato. The eyes were allowed to rest for 6 hours after IOP was applied and before the first imaging session. Using a multiphoton microscope (Nikon A1MP^+^, Nikon Instruments Inc, Melville, NY, USA) on day 1 of organ culture; one square pattern was photobleached into each scleral shell across multiple lamellae using a 25x objective (Nikon CFI75 Apo LWD 25XW, Nikon Instruments Inc, Melville, NY, USA), and multiple grid patterns were photobleached into individual scleral lamellae of each sclera using the same objective and the zoomed scan feature of the microscope at 60x magnification. SHG imaging was used in parallel to 2PFM of the CNA35-tdTomato labeled collagen to localize individual lamellae on day 1 of organ culture (Fig 3). Each pattern was photobleached for 5 seconds at 50% laser power using 700 nm laser wavelength. SHG imaging was performed at 800 nm laser wavelength. In a preliminary study, this laser power and exposure time were identified to photobleach clear patterns without causing thermal damage to the collagen structures (Fig 4). The photobleached patterns were imaged on days 1, 2, and 3 of organ culture. While the original patterns were photobleached within one xy-plane, these patterns deformed three-dimensionally as the scleral shell remodeled and were not within one xy-plane on consecutive imaging days. Consequently, z-stacks were taken on days 2 and 3 to localize the patterns in the remodeled scleral configuration. The experiment was stopped after 3 days in organ culture as the fluorescent signal and the photobleached lines started to fade.

### Quantifying Scleral Remodeling

**Fig 5:**
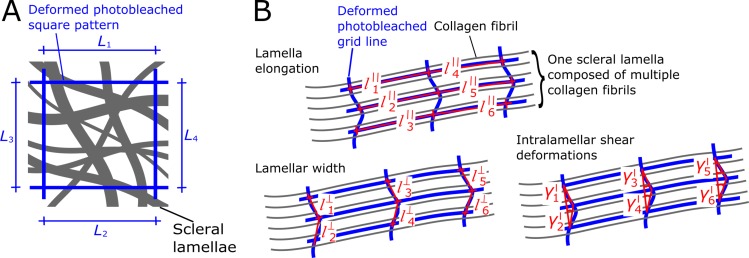
Schematic of photobleached patterns at the tissue- and lamella-level. (**A**) Photobleached square pattern (blue) showing the line segments (L_1_, L_2_, L_3_, and L_4_) used to quantify tissue-level elongations. (**B**) Photobleached grid pattern showing line segments used to quantify lamellar elongation ($l_{k}^{\|}$) and lamellar width changes ($l_{k}^{⊥}$), and shear angles ($\gamma_{k}^{l}$) used to quantify interlamellar deformations at the lamella-level.

Remodeling deformations were determined by analyzing the 2PFM images of the bleached patterns taken on days 1, 2, and 3 of organ culture. For each culture day and eye, we have manually delineated the corner and intersection points of the photobleached lamella- (6 points per lamellae) and tissue-level patterns (4 corner points). The three-dimensional coordinates of these points were used to calculate the grid pattern dimensions and how they deform in organ culture (Fig 5). At the tissue-level, tissue elongation (*ε*^t^) was calculated as the change in the photobleached square dimensions (*L*_1_ –*L*_4_) from day 1 to day *d* = 2, 3 in culture as:

$$\varepsilon_{i}^{t}(d)=\frac{L_{i}(d)}{L_{i}(1)}-1\;\text{for}\;i=1,\;2,\;3,\;4$$

At the lamella-level, we calculated the elongation of a lamella *l* ($\varepsilon_{k}^{l,\|}$) as the change in dimensions of the photobleached patterns parallel to the lamellar orientation $l_{k}^{\|}$ with *k* = 1, 2, 3, …6. Similar, we calculated lamellar width changes $\varepsilon_{k}^{l,⊥}$ as length changes of the photobleached pattern perpendicular to the lamellar orientation $l_{k}^{⊥}$.

$$\varepsilon_{k}^{l,\|}=\frac{l_{k}^{\|}(d)}{l_{k}^{\|}(1)}-1;\;\varepsilon_{k}^{l,⊥}=\frac{l_{k}^{⊥}(d)}{l_{k}^{⊥}(1)}-1$$

Intralamellar deformations were quantified as the shear angle $\gamma_{k}^{l}$ between the three points of the photobleached lines perpendicular to the lamellar orientation. As the definition of a positive shear angle direction is arbitrary, we decided to use the absolute value of $\gamma_{k}^{l}$ in our analyses.

The robustness of our scleral remodeling imaging and quantification method was evaluated by performing sample translation and rotation. After photobleaching and imaging on day 1, the bioreactor was removed from the microscope, the eye chambers were tilted by ~5º, and then returned to the microscope. The photobleached patterns were reimaged at their new three-dimensional locations. Tilting the eye chamber represents a rigid body motion, which should result in zero tissue- and lamella-level strains. To measure the robustness of our imaging and quantification methodology, we calculated the tissue- (*ε*^t^) and lamella-level deformations ($\varepsilon_{k}^{l,\|}$, $\varepsilon_{k}^{l,⊥}$, $\gamma_{k}^{l}$) after tilting the eye chambers. To test whether or not significant remodeling deformations occurred in organ culture, we tested for a significant difference of our tissue- and lamella-level measures and their variance with incubation days, where our robustness test represents the day 1 measurement for both eyes. For normally distributed variables, statistical significance of the mean and variance was evaluated using one-way ANOVA and Levene's test, respectively. For not normally distributed variables, statistical significance of our measurements and their variance was evaluated using the Kruskal-Wallis test (nonparametric ANOVA) and nonparametric Levene’s test, respectively. We considered the strain of each line segment ($\varepsilon_{i}^{t}$, $\varepsilon_{k}^{l,\|}$, and $\varepsilon_{k}^{l,⊥}$) and each shear angle measurement ($\gamma_{k}^{l}$) as an independent measurement in our statistical analysis.

## Results

### Viability Study

**Fig 6:**
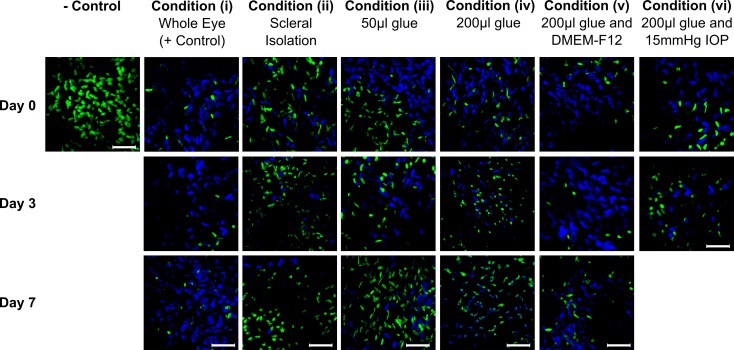
Cell viability in organ culture conditions (i-vi). Maximum intensity projections of representative z-stacks (40 μm) of each condition (i-vi) showing cell nuclei of all cells (Hoechst 33342;Blue) and dead cells (Sytox Green; Green) at day 0, 3, and 7 in culture, and negative control (tissue bathed in 70% methanol for 1h to kill all cells). The Sytox Green channel was overlaid on top of the Hoechst channel. Scale bars represent 50 μm.

**Fig 7:**
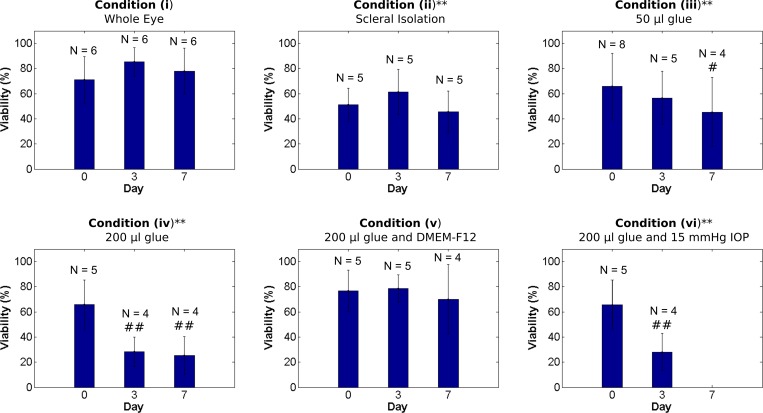
Bar graphs of cell viability in organ culture conditions (i-vi). * and ** indicate a significant difference in cell viability compared to the positive control condition (i) at the same day in organ culture with P < 0.05 and P < 0.001, respectively (unpaired t-test). # and ## indicate a significant reduction in cell viability compared to day 0 of the same condition with P < 0.05 and P < 0.001, respectively (unpaired t-test).

The results of the cell viability study are presented in Figs 6 and 7. The two-way ANOVA showed significant differences for each factor (condition, incubation days) and their interaction (p < 0.001). Post hoc analyses showed that cellular viability was not significantly altered throughout the culture period in the positive control condition (i) with minimal tissue manipulations: 71.0±18.6% (mean ± standard deviation) on day 0, 85.3±11.5% on day 3, and 78.1±2.11% on day 7. Compared to the control condition (i), all tissue manipulations (ii-iv) significantly lowered the average cell viability (p < 0.001). Removing all non-scleral tissues in condition (ii) significantly reduced the cell viability compared to the positive control. Condition (ii) showed no significant change in cell viability with incubation days: 51.2±13.0% on day 0, 61.4±18.1% on day 3, and 45.5±16.7% on day 7. Using 50 μL of glue to secure eyes to a stainless steel washer in condition (iii) significantly lowered the cell viability compared to positive control. Condition (iii) showed a significant reduction in cell viability with incubation days: 65.8±26.4% on day 0, 56.6±21.3% on day 3, and 45.4±27.7% on day 7. Using an increased amount of glue (200 μL) in condition (iv) further reduced the cell viability with incubation days: 65.8±19.4% on day 0, 28.2±11.8% on day 3, and 25.25±15.18% on day 7. Adding Ham’s F12 Nutrient Mixture in condition (v) offset the cytotoxic effect of glue resulting in cell viability that was not significantly different from the positive control (p = 0.581) and cell viability did not change with incubation days: 76.8±16.6% on day 0, 78.7±10.8% on day 3, and 73.0±38.5% on day 7. Culturing eyes in our bioreactor in condition (vi) resulted in cellular viability of 26.1±15.1% on day 3, which was similar to condition (iv) suggesting that IOP and media bubbling cannot offset the cytotoxic effect of glue (Figs 6 and 7).

### Scleral Remodeling in Organ Culture

**Fig 8:**
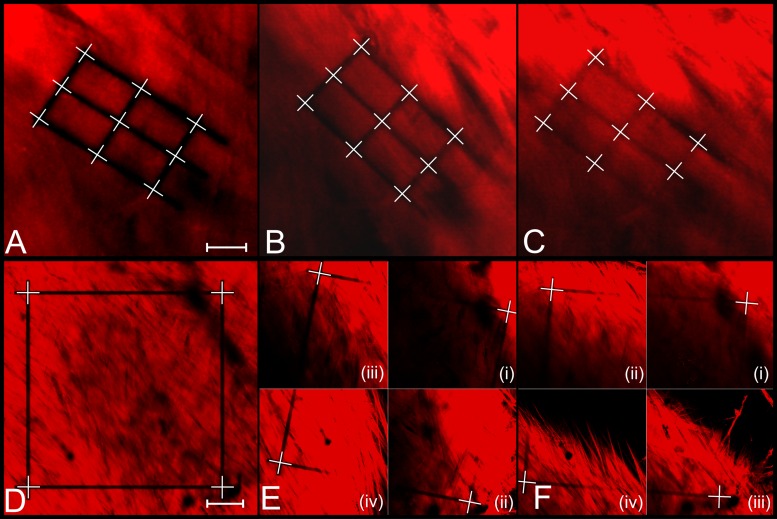
Representative 2PFM images of photobleached patterns at the lamella-level and tissue-level over three days in culture. Photobleached grid pattern of one representative lamella at the lamella-level (**A, B, C**) and photobleached square pattern at the tissue-level (**D, E, F**) on days 1 (**A, D**), 2 (**B, E**), and 3 (**C, F**) of organ culture. All images were taken from the right eye of tree shrew 1602 at 15 mmHg. Lamella-level images (**A, B, C**) were taken at ~60X and tissue-level images (**D, E, F**) were taken at 25X magnification. White crosshairs represent the manually delineated corner and intersection points of the grid patterns. Some lamella-level grid patterns partially faded at day 2 or 3 of organ culture as seen in **C**. Line segments related to points that could not be delineated (e.g. bottom right point in **C**) were excluded from the quantification of scleral remodeling deformations. **E** and **F** are composed of multiple images taken at different z-depths showing the three dimensional deformation of the square pattern as the sclera remodeled in organ culture. Image quadrants in **E** were taken at the following z-levels: (i) 2.275 μm, (ii) 12.025 μm, (iii) 18.200 μm, and (iv) 28.275 μm. Images quadrants in **F** were taken at the following z-levels: (i) 7.475 μm, (ii) 17.225 μm, (iii) 36.400 μm, and (iv) 46.150 μm. Scale bar in **A** represents 10 μm. Scale bar in **D** represents 50 μm.

Fig 8 shows one scleral lamella and one square pattern imaged after photobleaching on day 1 and reimaged on days 2 and 3. In total, one tissue-level square pattern was photobleached into each eye and 20 (13) grid patterns were photobleached into individual lamellae of the right (left) eye. During the culture period, the sclera deformed three-dimensionally and the photobleached squares had to be localized using z-stacks (Fig 8E and 8F). Some of the photobleached lamellar grids faded during the culture period and not all corner and intersection points could be delineated on days 2 and 3 (see Fig 8C). Line segments that connected to faded points were disregarded in the analysis.

**Fig 9:**
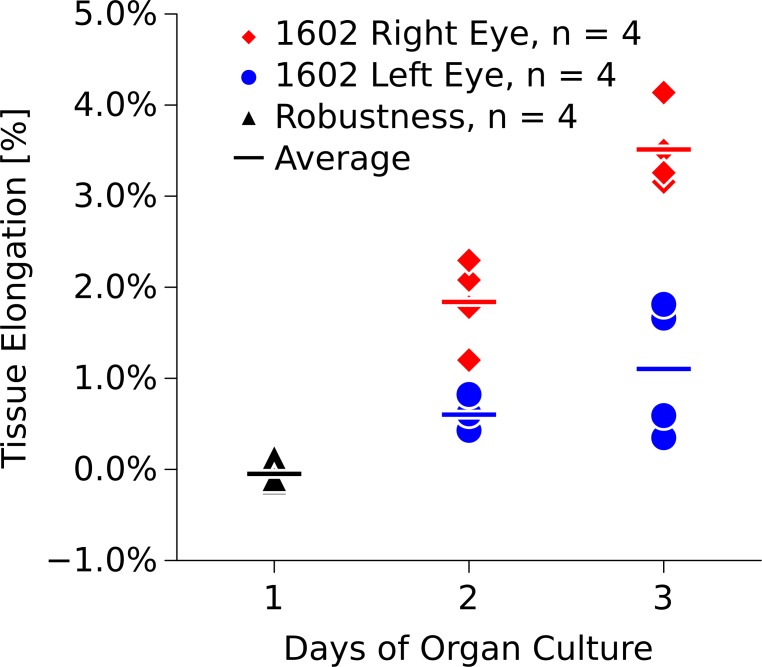
Tissue elongation in the left and right eye of tree shrew 1602 over three days in organ culture. Tissue elongation was measured as change in photobleached square dimensions for both eyes after 2 and 3 days in organ culture. The tissue-level strains significantly increased throughout the culture period (ANOVA, p < 0.001). Robustness values were measured after the eye chamber was tilted on day 1 of organ culture.

**Fig 10:**
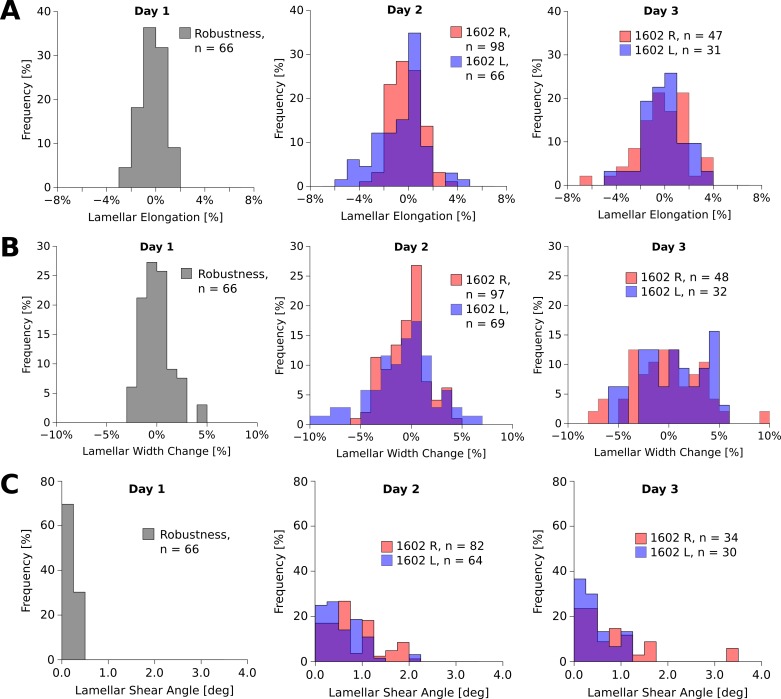
**Histograms of lamellar elongation (A), width change (B), and shear angle distributions (C) of the left (L) and right (R) eye of tree shrew 1602 over three days in organ culture.** The lamellar elongation (**A**) and width changes (**B**) were normally distributed around zero at all days of organ culture. The variance of the distributions significantly increased in organ culture (Levene’s test, P < 0.01). The shear angle (**C**) significantly increased in both eyes (Kruskal-Wallis test, P < 0.01). Robustness values were measured after the eye chamber was tilted on day 1 of organ culture.

Tables 1 and 2 summarize the results of the robustness test and remodeling deformations, respectively, at the tissue and lamellar levels. The number of independent measurements used in our statistical analysis is represented by the number of lines/angles listed in Tables 1 and 2. At the tissue-level, both scleras significantly elongated during the culture period (Fig 9) (ANOVA,P < 0.001). At the lamella-level, both, lamellar elongation and lamellar width changes were normally distributed (Fig 10A and 10B). The means of the lamellar elongation and lamellar width change distributions did not significantly change during incubation, while the variances significantly increased (Levene's test, P < 0.01) suggesting that some scleral lamellae may have elongated and others shortened during the culture period. The shear angle results were not normally distributed. The shear angle distribution and its variance significantly increased in organ culture in both eyes (Kruskal-Wallis and nonparametric Levene’s test, P < 0.01, Fig 10C).

**Table 1:** Robustness test results for tissue elongation, lamellar elongation, lamellar width changes, and lamellar shear angle from tree shrew 1602. The Number of Lines/Angles represents the number of independent measurements used for statistical analysis and to plot the histograms in Figs 9 and 10.

	Robustness (1602)						
Parameter	Number of Bleached Patterns	Number of Lines/Angles	Mean	Standard Deviation	Median	1^st^ Quartile	2^nd^ Quartile
**Tissue Elongation**	1	4	-0.05%	0.12%	-0.10%	-0.11%	-0.04%
**Lamellar Elongation**	11	66	-0.24%	0.92%	-0.12%	-0.87%	0.42%
**Lamellar Width Changes**	11	66	0.03%	1.50%	-0.04%	-1.23%	0.83%
**Shear Angle [deg]**	11	66			0.14	0.03	0.28

**Table 2:** Results for tissue elongation, lamellar elongation, lamellar width changes, and shear angle in the left and right eye of tree shrew 1602. The Number of Lines/Angles represents the number of independent measurements used for statistical analysis and to plot the histograms in Figs 9 and 10.

Parameter	Eye	Day	Number of Bleached Patterns	Number of Lines/Angles	Mean	Standard Deviation	Median	1^st^ Quartile	3^rd^ Quartile
**Tissue Elongation**	Left	2	1	4	0.60%	0.17%	0.58%	0.52%	0.66%
		3	1	4	1.10%	0.74%	1.13%	0.53%	1.70%
**Lamellar Elongation**		2	13	66	-0.67%	1.93%	-0.24%	-1.84%	0.55%
		3	6	31	-0.17%	1.78%	-0.05%	-1.46%	0.91%
**Lamellar Width Changes**		2	13	69	-0.83%	3.23%	-0.48%	-2.86%	1.09%
		3	6	32	0.46%	3.23%	0.64%	-1.84%	3.58%
**Shear Angle [deg]**		2	13	64			0.47	0.25	0.85
		3	6	30			0.28	0.14	0.59
**Tissue Elongation**	Right	2	1	4	1.84%	0.48%	1.93%	1.64%	2.13%
		3	1	4	3.51%	0.44%	3.38%	3.23%	3.66%
**Lamellar Elongation**		2	20	98	-0.16%	1.22%	-0.20%	-1.12%	0.63%
		3	12	47	-0.20%	2.01%	-0.10%	-1.32%	1.21%
**Lamellar Width Changes**		2	20	97	-0.50%	2.08%	-0.43%	-1.88%	0.72%
		3	12	48	-0.23%	3.42%	-0.29%	-2.69%	2.19%
**Shear Angle [deg]**		2	20	82			0.63	0.34	1.07
		3	12	34			0.62	0.27	1.12

## Discussion

We have presented the first scleral organ culture model that allows to account for IOP and use of serum-free culture media. We have demonstrated that tree shrew sclera can be cultured *in situ* for at least 7 days in serum free media without significant loss of cells. Culturing condition (ii) has revealed that tree shrew scleral fibroblasts are sensitive to tissue manipulations during tissue processing. Compared to human sclera, the tree shrew sclera is very thin (~100 m) and flexible. Inverting the scleral shell may have introduced high bending deformations. Removing the retina and choroid may have introduced additional shear strains and/or damaged the sclera. These mechanical manipulations may have caused the significant loss of scleral fibroblast in culturing condition (ii). Also, the intraocular tissues (retina, choroid, retinal pigment epithelium) may play an important role in supporting the viability of scleral fibroblasts and the removal of these tissues may have promoted scleral fibroblast loss in condition (ii). Culturing conditions (iii) and (iv) revealed a cytotoxic effect of cyanoacrylate adhesives on scleral fibroblasts. This cytotoxic effect was seen after three days in organ culture. Increasing the amount of glue from 50 (iii) to 200 *μ*l (iv) decreased the cell viability by about 50% on day 3 of organ culture suggesting a dose-dependent cytotoxic effect of cyanoacrylate. It is important to note that the increased amount of glue was important to ensure continues bonding of the eye to the stainless steel washer without leakage when placed into our bioreactor and subjected to IOP. We have tried to use bioadhesives (data not shown) to avoid the cytotoxic effect of cyanoacrylate but the tested bioadhesives were unable to maintain a permeant seal between the eye and the washer throughout the culture period.

The sclera is the main loadbearing collagenous tissue of the eye and continuously subjected to IOP *in vivo*. Therefore, scleral fibroblasts typically live in a mechanically strained ECM *in vivo*. It was previously shown that IOP alterations and cyclic mechanical strain can alter scleral fibroblast function [52, 53] and promote differentiation into myofibroblast [54]. Mechanical strain was also shown to promote fibroblast proliferation and survival [55, 56]. These findings support the notion that IOP and strain may have protective effects on fibroblast survival. This protective effect was not seen in our experiments as constant IOP had no significant impact on the cell viability (conditions (iv) versus (vi)) and was unable to reduce the cytotoxic effect of the cyanoacrylate.

The active ingredient of the glue (Permabond 792) used in our organ culture experiments is ethyl 2-cyanoacrylate. Ethyl cyanoacrylate adhesives have been used for wound closure and other medical applications [57, 58] but are also known to introduce cytotoxic effects to cell cultures [59, 60]. In contrast to IOP (condition (vi)), supplementing our culture media with Ham’s F12 Nutrient Mixture (condition (v)) completely offset the cytotoxic effect introduced by the cyanoacrylate adhesive resulting in cell viability that was not significantly different to the positive control condition (i) throughout the entire culture period of 7 days. Ham’s F12 Nutrient Mixture is a rich source of amino acids, vitamins, and nucleosides, many of which are not present in DMEM. The immediate supply of these media supplements can facilitate cell growth, repair, and other metabolic cell functions, which may have protected the scleral fibroblasts against the cytotoxic effect of cyanoacrylate in our experiments. To maximize cell survival/activity, cell, tissue, and organ culture experiments often use culture media that is supplemented with serum at 1% to 10% [35–37]. Inducing myopia in tree shrews has been shown to significantly change several mRNA levels. The reported changes include transforming growth factors (TGF-β 1–3) and MMPs (MMP-2 and MMP-14) suggesting that growth factors and MMPs are critical components in scleral remodeling pathways during myopia development [21, 23, 61]. Any serum added to the culture media is likely to alter the scleral remodeling process in organ culture as serum is rich in growth factors and known to alter MMP biosynthesis [38]. Consequently, establishing a serum-free organ culture system as proposed here is critical when studying scleral remodeling in organ culture.

We have developed a new portable bioreactor, which allows us to culture two tree shrew eyes subjected to IOP and to image their collagen structures on a daily basis using 2PFM. Using a level sensor and a computer controlled pump system, we were able to maintain constant IOP throughout the culture period while circulating oxygen-rich media between the media reservoir and the eye chamber. The bioreactor was built on top of a baseplate allowing us to place the bioreactor inside an incubator and transport it to the microscope for daily imaging.

This is the first study that used a photobleaching technique based on 2PFM to quantify three-dimensional micro-deformations that occur over a period of 3 days in organ culture. We used the two eyes of one untreated (normal) juvenile tree shrew in our experiment. Previous animal studies suggest that scleral remodeling is actively controlled by an emmetropization process during this age [10–14]. In the two tested eyes, tissue-level deformations increased throughout the culture period. While most of scleral fibroblast population died during the experiment (75%), the tissue-level deformations progressed throughout the 3 culture days. Consequently, it is unclear if the sclera passively deformed (creeped) or the fibroblasts actively remodeled the sclera in our organ culture experiment. If scleral remodeling is a creep-like mechanism as suggested by McBrien et al. [29], scleral remodeling may continue once the creep rate was modulated by the fibroblasts. In this case, fibroblast activity would only be needed to accelerate or slow/stop the creep-like remodeling. On the other hand, if scleral remodeling requires continuous cellular activity, the measured deformations may be wholly, or in part, due to the lack of such cellular activity. It remains unclear if the here measure tissue-level deformations represent scleral remodeling as it occurs *in vivo*. Additional experiments are needed to clarify the mechanism that underlies scleral remodeling during emmetropization and myopia development.

The lamella-level deformations show a complex picture. On average, lamellar elongation and width changes basically remained zero in the two investigated eyes. However, the variance of both variables (lamellar elongation and width changes) increased on days 2 and 3 when compared to our robustness test suggesting that some lamellae elongated while other shortened. This observation may be explained by interlamellar sliding that occurred in organ culture, where tension was increased in some lamellae and decreased in others. The lamellar shear angle significantly increased with culture time in both eyes suggesting that interlamellar deformations such as micro-deformations between collagen fibrils may have occurred during the culture period. These micro-scale results may be due to relative deformations between collagen fibrils, which accumulate to lamellar and ultimately tissue-level elongations as previously suggested [30]. However, further studies are required to clarify this point.

### Limitations

It is important to consider the limitations of the presented *in situ* methodology for quantifying scleral collagen remodeling. *In vivo*, eyes receive varied signaling throughout the day simply due to alteration of focus and light intake. This affects the resultant signaling cascades from the retina to the sclera in ways that are currently not fully understood. The lack of this signaling in our organ culture system may have unknown effects on scleral remodeling.

Our bioreactor accounts for constant IOP. *In vivo*, IOP is not constant but fluctuates at very different frequencies [62, 63]. To which extent IOP and IOP fluctuations are important for scleral remodeling in myopia is unclear. Recently, we reported an increased cyclic softening response of scleral strips from tree shrews developing myopia [64] suggesting that cyclic forces may promote internal deformations such as scleral lamellae or collagen fibril sliding [30]. We are planning to advance our bioreactor to account for IOP fluctuations by adding an IOP feedback control system that would raise and lower the reservoirs.

The intraocular tissues remained in the eyes during the culture period. While the viability of the retinal pigment epithelium and retina were not investigated, these tissues likely died during the culture period. The signaling from apoptotic and necrotic cells may have affected scleral fibroblast signaling and function and/or scleral biomechanics in our experiment. Furthermore, our bioreactor necessitates the use of glue, and although its negative effect on cell viability was removed by using Ham’s F12 Nutrient Mixture, it may have altered cell signaling and scleral remodeling *in situ*.

Another limitation is that the time course of this experiment cannot be consistently extended past 3 days, and, therefore, limits our ability to study slower deformations. When extending the study past 3 days, bleached lines begin to disappear due to the reversible binding of the CNA35 protein [65]. Any photobleached pattern cannot reliably be imaged and segmented beyond 3 days.

It is important to note that scleral lamellae in tree shrews are very thin [19]. Despite the superior sectioning capability of 2PFM, emitted light of scleral lamellae that are below or above the imaging plane can been seen in our images (see Fig 3). Consequently, the here reported micro-deformations may be biased by interlamellar deformations have to be interpreted with caution. Furthermore, the CNA35 fluorescent signal was very bright and lamellae edges were not always clearly visible. In these cases, we used SHG imaging to localize scleral lamella and position the photobleached patterns on day 1 of organ culture (see also Fig 3).

Finally, the reported micro-deformations are based on measurements in only two eyes. Furthermore, the 2PFM study was performed before we discovered the protective effect of Ham's F-12 Nutrient Mixture. Consequently, the significant loss of scleral fibroblasts due to the cytotoxic effect of cyanoacrylate may have impacted our results. The experiment has to be repeated, Ham's F-12 Nutrient Mixture need to be added to the culture media, and scleras of myopia developing animals have to be measured to understand if the observed deformations are due to scleral remodeling.

## Conclusions

The results of this study show that tree shrew scleral shells can be kept alive for up to 7 days in serum-free culture media, and with the proper media formulation, can be kept alive without significant loss of cell viability even after the addition of cyanoacrylate adhesive necessary for placement in our bioreactor. Our bioreactor allows us to keep the sclera alive, apply static IOP, and transport the eyes to and from a 2PFM for photobleaching and daily imaging of scleral collagen. This is the first study that recorded micro-deformations in living scleral shells over a period of 3 days *in situ*.

## Supporting Information

S1 Table Tissue elongation data (tissue-level) used in Tables 1 and 2 and Fig 9. Day 1 values represent the data of our robustness test.
(CSV)

S2 Table Lamellar elongation data (lamella-level) used in Tables 1 and 2 and Fig 10. Day 1 values represent the data of our robustness test.
(CSV)

S3 Table Lamellar width data (lamella-level) used in Tables 1 and 2 and Fig 10. Day 1 values represent the data of our robustness test.
(CSV)

S4 Table Lamellar shear angle data (lamella-level) used in Tables 1 and 2 and Fig 10. Day 1 values represent the data of our robustness test.
(CSV)
